# Charge-separated state trap: a new strategy for constructing high-performance organic scintillators

**DOI:** 10.1093/nsr/nwaf104

**Published:** 2025-03-20

**Authors:** Zhiyong Tang

**Affiliations:** CAS Key Laboratory of Nanosystem and Hierarchical Fabrication, CAS Center for Excellence in Nanoscience, National Center for Nanoscience and Technology, China

Owing to their inherent advantages, such as the absence of heavy metal components, highly designable synthetic properties, low raw material costs, and low usage costs, organic scintillators are considered to be the most promising scintillator materials [1]. In the 1940s, organic crystals such as anthracene and naphthalene were found to exhibit scintillation characteristics, thus becoming the earliest organic scintillators. In the 1960s, researchers initiated the utilization of plastic scintillators to increase both light output efficiency and stability. After the 1990s, inspired by organic light-emitting diode (OLED) technology, new types of conjugated organic semiconductors were developed, further enhancing the performance of organic scintillators [2,3].

Organic scintillators have achieved substantial advancements in the past several decades. However, since they are mainly composed of light elements, their absorption of high-energy rays is restricted, leading to generally lower radio-luminescence intensities than those of inorganic scintillators. In recent years, through the introduction of heavy halogen atoms and the property of thermally activated delayed fluorescence (TADF) into organic molecules, the issues of weak X-ray absorption and low exciton utilization efficiency of organic scintillators have been addressed to some extent [4,5]. Nevertheless, among the organic scintillators reported to date, only a very limited number exhibit remarkable performance, making it challenging to substitute the dominant position of inorganic scintillators in X-ray imaging technology.

**Figure 1.**
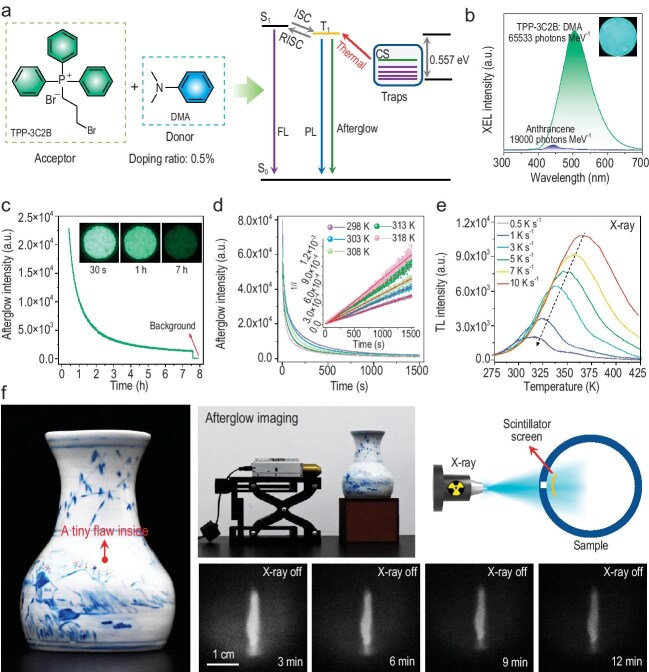
(a) Excited-state model of the TADP process induced by the CS state traps. (b) XEL spectra of TPP-3C2B: DMA and anthracene with the same thickness and cross-sectional area. Inset: photo of TPP-3C2B: DMA under X-ray. (c) Afterglow profile of TPP-3C2B: DMA after X-ray excitation. Inset: photos of X-ray excited afterglow. (d) Time-dependent emission decay curves of TPP-3C2B: DMA at different temperatures after X-ray excitation was stopped. Inset: the curves were fitted with the second-order reaction dynamics to calculate *k*. (e) Thermoluminescence spectra of TPP-3C2B: DMA after X-ray excitation at different heating rates. (f) X-ray afterglow imaging based on flexible organic scintillation films. Reprinted from Ref. [6].

Recently, Qiu-Chen Peng, Kai Li, Shuang-Quan Zang and their co-workers proposed a brand-new design strategy for significantly enhancing the

 radio-luminescence intensity of organic scintillators. In their work, they developed an organic scintillator by doping the electron-donating molecule, *N, N*-dimethylaniline (DMA), with the electron-withdrawing compound TPP-3C2B (TPP-3C2B: DMA), and constructed a charge-separation (CS) state trap within this doping system for the effective capture of high-energy electrons (Fig. 1a) [6]. The thermally activated delayed phosphorescence (TADP) mechanism induced by the CS state endow the organic scintillators with a relative light yield (LY) as high as 65 533 photons MeV^−1^ (Fig. 1b). Additionally, the presence of the CS state trap has enabled organic scintillators to achieve X-ray–induced ultralong room-temperature afterglow characteristics for the first time (Fig. 1c).

In the mechanistic investigation, two complementary approaches were adopted to reveal the existence of the CS state. First, by fitting the variable-temperature afterglow decay curves to the second-order reaction kinetics and using the Arrhenius equation, the activation energy for the relaxation of excitons from the trap state to the luminescent state was determined (Fig. 1d). Second, using thermoluminescence spectroscopy and the Hoogenstraaten method, the trap depth of the CS state was obtained (Fig. 1e).

The authors utilized the flexible scintillation film fabricated from the organic scintillator to develop X-ray afterglow imaging technology at room temperature. This technology is capable of effectively detecting the internal structural defects of objects and provides a new approach for industrial non-destructive flaw detection and the identification of cultural relics (Fig. 1f). Overall, this work innovatively proposes a strategy for capturing high-energy carriers through excited-state traps and can be used to facilitate the design and synthesis of high-performance organic scintillators.
